# Bioactive Oligopeptides from Ginseng (*Panax ginseng* Meyer) Suppress Oxidative Stress-Induced Senescence in Fibroblasts via NAD^+^/SIRT1/PGC-1α Signaling Pathway

**DOI:** 10.3390/nu14245289

**Published:** 2022-12-12

**Authors:** Na Zhu, Mei-Hong Xu, Yong Li

**Affiliations:** 1 Department of Nutrition and Food Hygiene, School of Public Health, Peking University, Beijing 100191, China; 2 Department of Nutrition and Food Hygiene, College of Public Health, Inner Mongolia Medical University, Hohhot 010059, China; 3 Beijing Key Laboratory of Toxicological Research and Risk Assessment for Food Safety, Peking University, Beijing 100191, China

**Keywords:** ginseng oligopeptides, senescence, oxidative stress, inflammation, mitochondrial function

## Abstract

The physicochemical properties and multiple bioactive effects of ginseng oligopeptides (GOPs), plant-derived small molecule bioactive peptides, suggest a positive influence on health span and longevity. Given this, cellular senescence is the initiating factor and key mechanism of aging in the organism, and thus the current study sought to explore the effects of GOPs on H_2_O_2_-induced cellular senescence and its potential mechanisms. Senescence was induced in mouse embryonic fibroblasts NIH/3T3 by 4 h of exposure to 200 µM H_2_O_2_ and confirmed using CCK-8 assay and Western blot analyses of p16^INK4A^ and p21^Waf1/Cip1^ after 24 h of growth medium administration with or without GOPs supplementation (25, 50, and 100 µg/mL). We found that GOPs delayed oxidative stress-induced NIH/3T3 senescence by inhibiting the G1 phase arrest, increasing DNA synthesis in the S phase, decreasing the relative protein expression of p16^INK4A^ and p21^Waf1/Cip1^, promoting cell viability, protecting DNA, and enhancing telomerase (TE) activity. Further investigation revealed that the increase in antioxidative capacity and anti-inflammation capacity might form the basis for the retarding of the senescence effects of GOPs. Furthermore, GOPs supplementation significantly improved mitochondrial function and mitochondrial biogenesis via the NAD^+^/SIRT1/PGC-1𝛼 pathway. These findings indicate that GOPs may have a positive effect on health span and lifespan extension via combating cellular senescence, oxidative stress, and inflammation, as well as modulating longevity regulating pathway NAD^+^/SIRT1/PGC-1𝛼.

## 1. Introduction

Aging is characterized as a time-dependent functional impairment that contributes to increased vulnerability to multiple human pathologies and death [1]. Organisms adapt and respond to the surrounding nutrient sources, and their various life activities are regulated by a network of nutrients and nutrient-sensing pathways [2]. The latest evidence suggests that the restriction of dietary energy, protein, or amino acids, can expand lifespan, improve metabolic disorders, and reduce the risk of aging-related diseases [3]. However, an observational study has shown that a high-protein diet is associated with a substantial reduction in cancer prevalence and all-cause mortality in populations over 65 years old [4]. Physiological changes occur with aging, such as decreased appetite, sensory loss, dysphagia, masticatory dysfunction, and gastrointestinal disorder, resulting in decreased food and energy intake. Although energy demands decrease with age due to a lower basal metabolic rate, the need for protein increases to make up for the age-related loss of skeletal muscle mass and function [5]. Dietary protein is a major source of amino acids. Reduced protein synthesis and catabolism of other amino acids result from an inadequate supply of amino acids [3]. Studies have shown that animals fed diets absent or deficient in essential amino acids had significantly shorter average survival times [6]. Several clinical studies have also shown that amino acid supplementation reduces sarcopenia and adjusts body fat and glucose metabolism in the aging population [7]. Actually, researchers suggest that middle-aged and elderly people should increase their daily protein intake above the recommended level [8,9].

Ginseng is a traditional Chinese medicinal herb and is commonly used for body-strengthening functions in Asian countries [10]. According to recent phytochemical and pharmacological studies, ginseng contains a variety of active ingredients, including ginsenosides, alkaloids, phenols, phytosterols, carbohydrates, peptides, ginseng oil, amino acids, micronutrients, and certain enzymes. The major functional ingredients are ginsenosides [11]. Ginseng has a wide range of pharmacological effects, is active in combating fatigue, hyperglycemia, obesity, and cancer, and possesses antioxidant, anti-inflammatory, and antiaging properties [12]. Ginseng has historically been assumed to extend longevity. Recent investigations have shown that ginseng components can increase the lifespan of experimental models like *Drosophila* and *Caenorhabditis elegans* [13,14]. Ginseng oligopeptides (GOPs) are obtained from *Panax ginseng* Meyer (ginseng) by bio-enzymatic digestion technology. Bioactive peptides can be used as protein substitutes in the daily diet to meet the amino acid requirements of the elderly population. However, the additional health benefits of bioactive peptides should be considered more severely. GOPs have a wide range of properties of bioactive peptides. Previous studies in our laboratory have confirmed the antioxidant [15], antifatigue [16], immunomodulatory [17], and pancreatic dysfunction-improving [18] activities of GOPs. These characteristics prompted us to focus on the potential of GOPs to extend health span and longevity. Furthermore, GOPs are plant-derived peptides that may play important role in inhibiting the progression of aging-related diseases. There is substantial evidence that plant-based proteins decrease the risk of diseases such as obesity, cardiovascular disease, type 2 diabetic disease, high blood pressure, and high-circulating cholesterol [3]. Higher intake of plant protein was also associated with lower all-cause mortality [2]. Bioactive peptides have a wide prospect in functional foods and medical applications. The food-derived bioactive peptides are highly safe, quickly absorbed, nontoxic, and have high protein and low fat characteristics compared with dietary proteins [19]. These exogenous peptides may function similarly to endogenous peptides, and some of them even affect the levels of endogenous active peptides and play a synergistic role with endogenous active peptides [20]. Overall, bioactive peptides should be considered excellent alternatives for antiaging treatment. Therefore, the purpose of present study was to investigate, for the first time, the delaying aging effects of GOPs at the cellular level and their possible mechanisms.

## 2. Materials and Methods

### 2.1. Chemicals

The GOPs sample was supplied by Jilin Taigu Biological Engineering Co., Ltd. (Jilin, China). It was derived from the roots of *Panax ginseng* C.A. Meyer by enzymatic hydrolysis as described previously [15]. The molecular weight of GOPs between 100 to 1000 Dalton accounted for 95.42%, and that of free amino acids amounted for 3.94%. The detailed information has been shown in Table 1.

### 2.2. Cell Culture and Treatments

Mouse embryonic fibroblast NIH/3T3 was purchased from the American Type Culture Collection (ATCC, Manassas, VA, USA). NIH/3T3 was cultured in Dulbecco’s Modification of Eagle’s Medium (DMEM) (GIBCO, Grand Island, NY, USA) containing 10% fetal bovine serum (Gibco, Grand Island, NY, USA) and 1% antibiotic-antimitotic (Coolaber, Beijing, China) at 37 °C under a humidified atmosphere of 5% CO_2_.

The H_2_O_2_-Induced NIH/3T3 senescent model was established in a manner previously described. NIH/3T3 was incubated for 4 h in a growth medium supplemented with different concentrations of H_2_O_2_ (from 50 to 800 µM) and then cultured in a growth medium for 24 h. The cell viability and the relative protein expression of p16^INK4A^ and p21^Waf1/Cip1^ were estimated to screen the effective intervention dose of H_2_O_2_.

The present research established a total of 5 groups: the control group and model group; and the low-, middle-, and high-dose GOPs groups. The control group was seeded in a growth medium. The model group was cultured in a growth medium with 200 µM of H_2_O_2_ for 4 h and then incubated in a growth medium without H_2_O_2_ for 24 h. The three GOPs administration groups were cultured in a growth medium supplemented with 200 µM of H_2_O_2_ and GOPs (25, 50, and 100 µg/mL, respectively), and the medium was removed after 4 h and then cultured in a H_2_O_2_ free growth medium containing 25/50/100 µg/mL of GOPs. The cells were collected for further investigation after being exposed to GOPs.

### 2.3. Cell Viability Assay

Cell viability was assayed using the cell-counting kit-8 (CCK-8) assay (KeyGEN, Nanjing, China) according to the manufacturer’s protocol. Briefly, about 1 × 10^4^ cells/well were added to 96-well plates. After treatment according to the protocol, 10 μL of CCK-8 was added to each well and cultured at 37 °C for 1–4 h. The absorbance ratio of each well was measured at 450 nm with a microplate reader (BMG FLUOstar Omega, Germany).

### 2.4. Flow Cytometry

Cells at 2 mL/well (about 2 × 10^5^) were seeded in 6-well plates and treated according to the protocol. For the cell cycle analysis, cells were harvested and washed twice with phosphate-buffered saline (PBS) and then fixed with 75% ethanol overnight at 4 °C. Following this, cells were washed three times with PBS and incubated with propidium staining and RNase A (Beyotime, Shanghai, China) for 30 min at 37 °C and analyzed using a Flow Cytometer (Beckman Coulter, Brea, CA, USA). For intracellular reactive oxygen species (ROS) analysis, cells were harvested and washed once with PBS, incubated for 20 min at 37 °C with the 10 μM of 2,7-dichlorofluorescein diacetate (Beyotime, Shanghai, China). After being washed with PBS three times, the cells were analyzed using a Flow Cytometer (Beckman Coulter, Brea, CA, USA). For mitochondrial membrane potential (∆Ψm) analysis, cells were harvested and stained with 500 µL of 1 × JC-1 dye solution (Beyotime, Shanghai, China) at 37 °C for 20 min in the dark. Then, the cells were washed twice and resuspended by 1 × JC-1 staining buffer. The change of fluorescence color was analyzed using flow cytometry (Beckman Coulter, Brea, CA, USA).

### 2.5. Biochemical Analysis

Cells at 2 mL/well (about 2 × 10^5^) were seeded in 6-well plates and treated according to the protocol. Then, the supernatant was obtained for the measurements of malondialdehyde (MDA) (Nanjing Jiancheng, Nanjing, China), glutathione peroxidase (GSH-Px) (Nanjing Jiancheng, Nanjing, China), superoxide dismutase (SOD) (Nanjing Jiancheng, Nanjing, China) activities, NAD^+^/NADH (Beyotime, Shanghai, China), telomerase (TE) activity (FEIYA, Jiangsu, China), interleukin-6 (IL-6) (Invitrogen, Waltham, MA, USA), IL-1β (Invitrogen, Waltham, MA, USA), matrix metalloproteinase-3 (MMP-3) (Multisciences, Hangzhou, China), intercellular cell adhesion molecule-1 (ICAM-1) (Multisciences, Hangzhou, China), and vascular cell adhesion molecule-1 (VCAM-1) (Multisciences, Hangzhou, China) using commercial kits.

### 2.6. Western Blot Analysis

Cells at 2 mL/well (about 2 × 10^5^) were seeded in 6-well plates and treated according to the protocol. Cells were collected and washed twice with PBS and then resuspended in RIPA Lysis Buffer (Biosharp, HeFei, China) supplemented with 1 mM of phenylmethanesulfonyl fluoride. Protein was extracted by centrifugation at 14,000× *g* for 15 min at 4 °C, and the concentration of protein was measured with a BCA protein assay kit (Thermo Scientific, Waltham, MA, USA). Equal amounts of protein (80–150 μg) were separated by 10–20% SDS-PAGE gel and transferred to PVDF membranes at different electric currents according to the size of protein molecules. The membranes were blocked for 2 h in 5% nonfat milk dissolved with Tris-buffered saline containing 0.05% Tween-20 (TBST) at room temperature. Protein expression was detected using a primary antibody p16^INK4A^ (1:1000, CST, Danvers, MA, USA), p21^Waf1/Cip1^ (1:1000, CST, Danvers, MA, USA), γ-H2A.X (1:1000, Abcam, Cambridge, MA, USA), AMPK𝛼 (1:1000, CST, Danvers, MA, USA), PGC-1𝛼 (1:2000, Abcam, Cambridge, MA, USA), SIRT1 (1:1000, CST, Danvers, MA, USA), β-actin (1:5000, Abcam, Cambridge, MA, USA), and horseradish peroxidase-conjugated antirabbit secondary antibodies (1:10,000, Abcam, Cambridge, MA, USA). Quantitative analysis of Western blot was performed using Image-Pro Plus (Media Cybernetics, Rockville, MD, USA).

### 2.7. Statistical Analysis

Statistical analyses were performed using the SPSS software version 24 (SPSS Inc., Chicago, IL, USA). Data are expressed as mean ± standard deviation (SD) and analyzed by one-way analysis of variance (ANOVA) test; to analyze the difference of parametric samples among groups, multiple comparisons of least significant difference (equal variances assumed) or Dunnett’s T3 test (equal variances not assumed) was used. *p* < 0.05 indicated a statistically significant difference.

## 3. Results

### 3.1. Effect of GOPs on Hallmarks of NIH/3T3 Senescence

**Figure 1:**
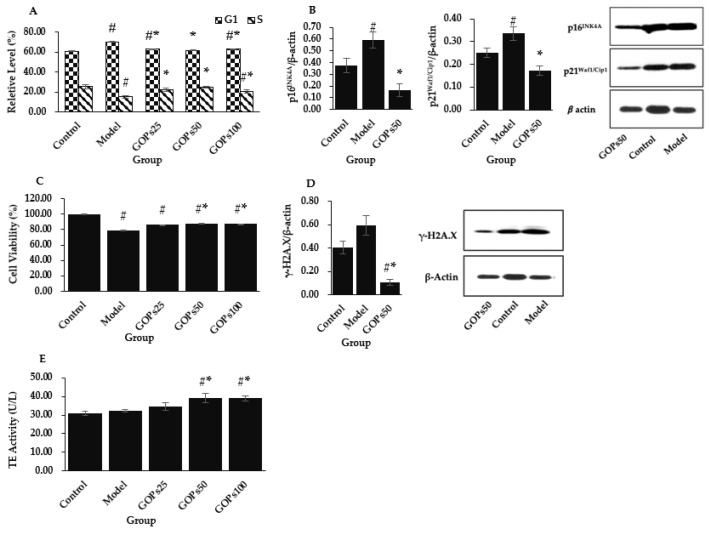
Effect of ginseng oligopeptides (GOPs) on the hallmarks of senescence in NIH/3T3. (**A**) Effect of GOPs on the cell cycle distribution of the NIH/3T3 cells (*n* = 3 per group). (**B**) Effect of GOPs on the relative protein expression of p16^INK4A^ and p21^Waf1/Cip1^ (*n* = 3 per group). (**C**) Cell viability evaluation of GOPs using the CCK-8 assay (*n* = 4 per group). (**D**) Effect of GOPs on DNA damage (*n* = 3 per group). (**E**) Effect of GOPs on telomerase (TE) activity (*n* = 3 per group). Values represented the mean ± S.D. # *p* < 0.05 versus control group, * *p* < 0.05 versus model group.

Cell cycle analyses indicated that aggravated oxidative stress induced partial G1 arrest in NIH/3T3 as evidenced by a higher percentage of cells in the G1 phase along with the concurrent decreases of the S fractions (*p* < 0.05). Compared with the model group, GOPs administration significantly inhibited oxidative stress-induced cell cycle arrest in three GOPs treated groups as reflected by a lower percentage of cells in the G1 phase and a higher percentage of cells in the S phase (*p* < 0.05) (Figure 1A). We further analyzed the relative protein expression level of p16^INK4A^ and p21^Waf1/Cip1^, and they were obviously upmodulated in the model group (*p* < 0.05). Meanwhile, this phenomenon was suppressed with the supplementation of GOPs with respect to the model group (*p* < 0.05) (Figure 1B).

CCK-8 cell viability assay was applied to measure cell viability, and decreased cell viability was observed in four H_2_O_2_-treated groups (*p* < 0.05). Compared with the model group, the cell viability was both significantly upmodulated in the 50 and 100 µg/mL of GOPs administration groups (*p* < 0.05) (Figure 1C).

γ-H2A.X was used as a reference marker for DNA damage. Enhanced DNA damage as a consequence of the exogenous administration of H_2_O_2_ was observed in the model group compared with the control group (*p* > 0.05). Compared with the control group, the relative protein expression of γ-H2A.X was significantly decreased in the 50 µg/mL of GOPs supplementation group (*p* < 0.05) (Figure 1D).

Regarding TE activity, no obvious changes were found between the control and model groups (*p* > 0.05). However, the TE activity was significantly enhanced in the 50 and 100 µg/mL of GOPs supplementation groups compared with the control group and the model group (*p* < 0.05) (Figure 1E).

### 3.2. Effect of GOPs on Oxidative Stress Status

**Figure 2:**
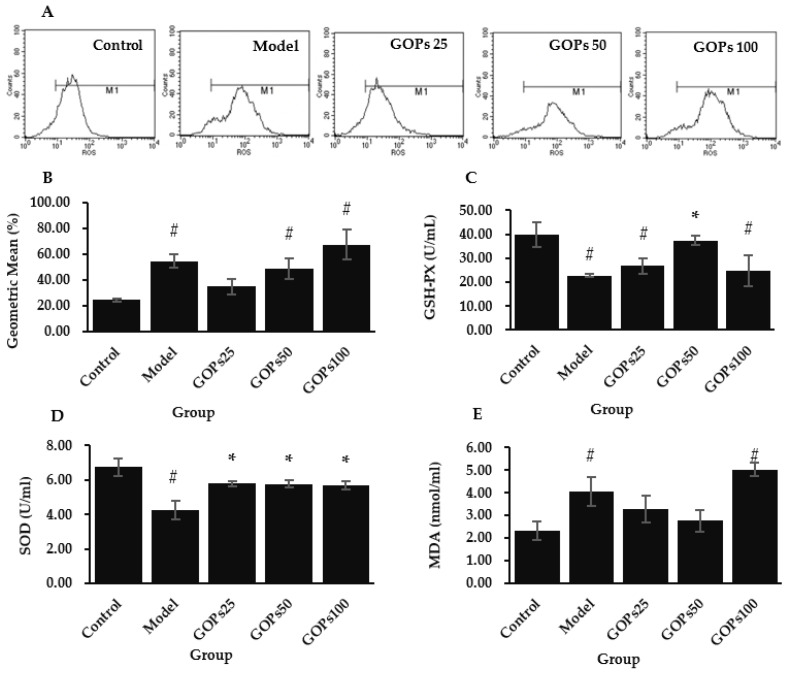
Effect of GOPs on the oxidative stress status of NIH/3T3. (**A**) Effect of GOPs on the fluorescence intensity of reactive oxygen species (ROS). (**B**) Quantitative analysis of ROS fluorescence intensity. (**C**) Effect of GOPs on glutathione peroxidase (GSH-Px) activity. (**D**) Effect of GOPs on superoxide dismutase (SOD) activity. (**E**) Effect of GOPs on malondialdehyde (MDA) levels. Values represent the mean ± S.D. (*n* = 3 per group). # *p* < 0.05 versus control group, * *p* < 0.05 versus model group.

The evaluation of oxidative stress status was completed with assessment of intracellular ROS, GSH-Px, SOD, and MDA. Compared with the control group, the intracellular ROS level in the model group was significantly increased (*p* < 0.05). Compared with the model group, the ROS generation tended to decrease in the 25 and 50 µg/mL of GOPs supplementation groups (*p* > 0.05); most notably, the 25 µg/mL of GOPs administration group returned the ROS content to the basal level (*p* > 0.05) (Figure 2A,B).

The antioxidant enzyme activities were inhibited in oxidative stress-induced senescent cells. Compared with the control group, GSH-Px and SOD activity was dramatically decreased in the model group (*p* < 0.05). Compared with the model group, the 50 µg/mL of GOPs supplementation group significantly enhanced GSH-Px activity (*p* < 0.05), while three concentrations of GOPs groups all markedly boosted SOD activity (*p* < 0.05) (Figure 2C,D).

MDA is the main metabolite of lipid peroxidation. Further analysis showed that the MDA concentration in the model group was significantly increased with respect to the control group (*p* < 0.05). Compared with the model group, the 25 and 50 µg/mL of GOPs supplementation groups tended to decrease MDA production to the basal level (*p* > 0.05) (Figure 2E).

### 3.3. Effect of GOPs on Senescence-Associated Secretory Phenotype (SASP)

**Figure 3:**
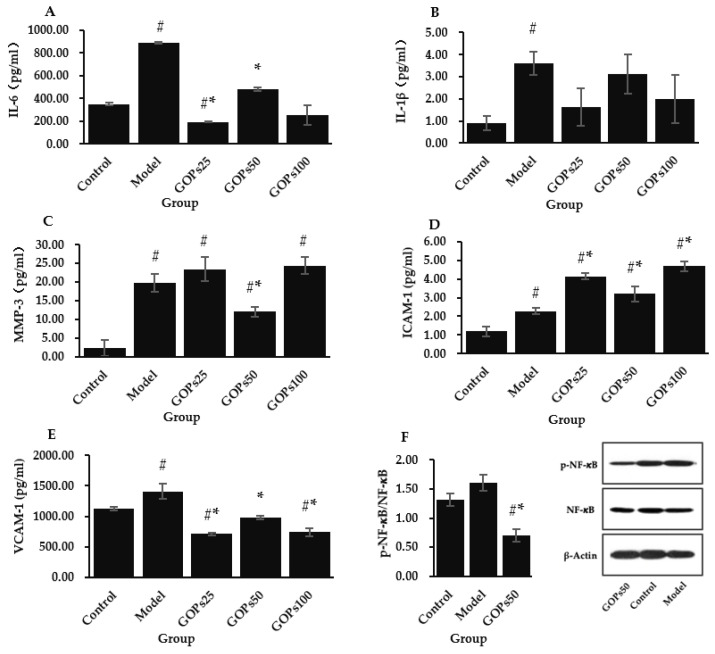
Effect of GOPs on the senescence-associated secretory phenotype (SASP). (**A**) Effect of GOPs on the interleukin-6 (IL-6) concentration. (**B**) Effect of GOPs on the IL-1β concentration. (**C**) Effect of GOPs on the matrix metalloproteinase-3 (MMP-3) concentration. (**D**) Effect of GOPs on the intercellular cell adhesion molecule-1 (ICAM-1) concentration. (**E**) Effect of GOPs on the vascular cell adhesion molecule-1 (VCAM-1) concentration. (**F**) Effect of GOPs on the activation of p-NF-κB/NF-κB. Values represent the mean ± S.D. (*n* = 3 per group). # *p* < 0.05 versus control group, * *p* < 0.05 versus model group.

The production of IL-6, IL-1, MMP-3, ICAM-1, and VCAM-1 was tested to determine the impact of GOPs on the senescent NIH/3T3 inflammatory phenotype (Figure 3A–E). Compared with the control group, the concentrations of IL-6, IL-1β, MMP-3, ICAM-1, and VCAM-1 were significantly higher in the model group (*p* < 0.05). Compared with the model group, GOPs supplementation groups tended to decrease IL-1β secretion to the normal level (*p* > 0.05), while 25 and 50 µg/mL of GOPs supplementation groups dramatically reduced IL-6 secretion (*p* < 0.05). GOPs administration groups also significantly decreased ICAM-1 concentration (*p* < 0.05), and 50 µg/mL of GOPs supplementation group significantly inhibited MMP-3 secretion (*p* < 0.05). One unanticipated finding was that ICAM-1 level significantly increased in the three GOPs administration groups compared with the control group and model group (*p* < 0.05).

To explore the anti-inflammatory mechanism of GOPs, we examined the effect of GOPs on the markers of the NF-κB pathway. NF-κB is an important mediator for cellular responses to inflammatory stimuli. Compared with the control group, H_2_O_2_ intervention tended to enhance the activation of NF-κB (p-NF-κB/NF-κB) in the model group (*p* > 0.05). Compared with the model group, the activation of NF-κB strongly attenuated in the 50 µg/mL of GOPs supplementation group (*p* < 0.05) (Figure 3F).

### 3.4. Effect of GOPs on Mitochondrial Function and Biogenesis

**Figure 4:**
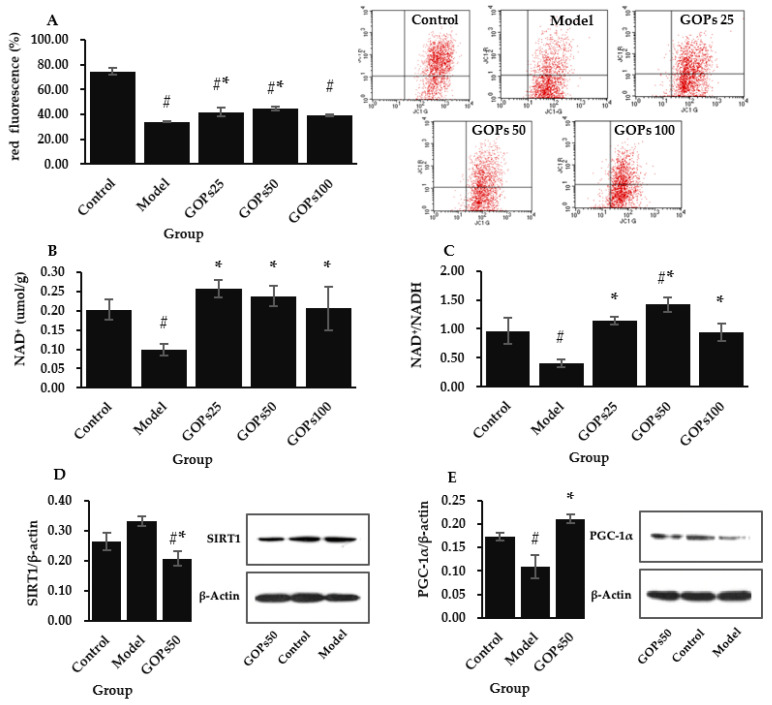
Effect of GOPs on mitochondrial function and biogenesis in NIH/3T3. (**A**) Effect of GOPs on the mitochondrial membrane potential; (**B**) Effect of GOPs on the NAD^+^ levels; (**C**) Effect of GOPs on the NAD^+^/NADH; (**D**) Effect of GOPs on the relative protein expression of SIRT1. (**E**) Effect of GOPs on the relative protein expression of PGC-1𝛼. Values represented the mean ± S.D. (*n* = 3 per group). # *p* < 0.05 versus control group, * *p* < 0.05 versus model group.

Loss of mitochondrial membrane potential and impairment of mitochondrial bioenergetics was detected in senescent NIH/3T3 cells. Compared with the control group, mitochondrial membrane potential declined considerably in the model group (*p* < 0.05). Compared with the model group, mitochondrial membrane potential was significantly enhanced in the 25 and 50 µg/mL of GOPs supplementation groups (*p* < 0.05) (Figure 4A). We further analyzed the effect of GOPs on mitochondrial biogenesis signaling NAD^+^/SIRT1/PGC-1𝛼. Compared with the control group, NAD^+^ concentration and NAD^+^/NADH both significantly decreased in the model group (*p* < 0.05). Compared with the model group, NAD^+^ levels and NAD^+^/NADH were all significantly upmodulated in the different concentrations of GOPs administration groups (*p* < 0.05) (Figure 4B,C). Compared with the control group, the relative protein expression of SIRT1 tended to increase in the model group (*p* > 0.05). Moreover, the relative protein expression of SIRT1 was greatly decreased in the 50 µg/mL of GOPs supplementation group compared with the control group and the model group (*p* < 0.05) (Figure 4D). Compared with the control group, the relative protein expression of PGC-1𝛼 was significantly downregulated in the model group (*p* < 0.05). Compared with the model group, the relative protein expression of PGC-1𝛼 was significantly increased in the 50 µg/mL of GOPs treated group (*p* < 0.05) (Figure 4E).

## 4. Discussion

There is a growing body of research demonstrating the effectiveness of bioactive peptides in extending lifespan and delaying aging. GOPs are plant-derived protein hydrolysates with a distinct amino acid pattern and lower rates of methionine and branched-chain amino acids, which are linked to a shortened lifespan. Several scientific studies in our laboratory suggest that GOPs may have a positive influence on prolonging a healthy lifespan. We attempted to probe the implication of GOPs on the aging process and their potential mechanisms from the cellular level through a series of experiments. Therefore, in the present work, a biological senescence model was established by culturing NIH/3T3 in vitro, and then the effect of GOPs on cell senescence was investigated. Many researchers have utilized mouse fibroblast NIH/3T3 cells to measure cellular aging and identify aging-related changes in the organism [21,22,23]. One significant mechanism of aging is the accumulation of senescent cells, which can be characterized as a steady arrest of the cell cycle caused by telomere shortening [24]. There are other aging-associated incitants, such as oxidative stress, ionizing radiation, and nutritional imbalance, trigger senescence independently of the telomeric process, which is known as premature senescence [25]. Among the contributing factors, ROS-induced oxidative stress is one of the most important [4]. Thus, to replicate naturally senescent cells, the oxidative-induced premature senescence model is frequently employed in scientific research [26,27,28], including the current study.

Multiple indicators were employed to assess cell senescence in the current study. We found that 4 h of 200 µM H_2_O_2_ treatment to NIH/3T3 successfully blocked the cell cycle in the G1 phase and decreased the proportion of cells in the S phase in contrast to the control group. Cyclin-dependent kinase (CDK) and cyclin-dependent kinase inhibitor (CDKI) control cell cycle progression. The main driving factor of cell cycle arrest during aging is the CDKI encoded in the CDKN2A (p16^INK4A^) and CDKN1A (p21^Waf1/Cip1^) loci. We further found that the protein expression of both was significantly upmodulated in oxidative stress-induced senescent NIH/3T3. Synchronously, senescent NIH/3T3 exhibited an accelerated loss in cell viability and proliferation. Irreparable DNA damage can induce senescence [29], and γ-H2A.X is considered a biomarker of DNA damage [30]. It was found that 200 µM of H_2_O_2_ treatment tended to accelerate DNA damage. However, no significant reduction in the TE activity, which is needed to replicate completely the terminal ends of linear DNA molecules [31], was found in the comparison with normal NIH/3T3. We report first time that GOPs postponed oxidative stress-induced senescence of NIH/3T3. GOPs supplementation significantly inhibited cell cycle arrest and promoted DNA synthesis in the S phase. Further research revealed that one essential mechanism for this phenomenon may be correlated with the inhibition of p16^INK4A^ and p21^Waf1/Cip1^ expression in NIH/3T3. Bioactive peptides are known to enhance cell proliferation [32,33]. Consistent with the literature, this study found that GOPs promote cell viability in senescent NIH/3T3. This also accords with our earlier observations, in which GOPs supplementation greatly increased mouse spleen lymphocyte proliferation [17]. In addition, GOPs can protect DNA against oxidative stress in NIH/3T3. Given the one common contributor of aging is the overabundance of genetic damage throughout life [34], stabilization of genomic homeostasis by GOPs is greatly important for life extension. Surprisingly, GOPs significantly increased TE activity in NIH/3T3, indicating the capacity to counter telomere shortening. Taken together, these findings strengthen the hypothesis that GOPs may have a positive influence on prolonging healthy lifespan by delaying cellular senescence. Indeed, several researchers have used cellular and animal models, as well as human clinical trials, to demonstrate that bioactive peptides isolated from marine food, such as sea cucumber, sepia esculenta, herring milt, have antiaging properties [35].

There is compelling evidence that oxidative stress contributes to a variety of aging pathologies [36], and one essential strategy for combating the aging process is mitigating the levels of oxidative damage. We showed that GOPs possess a moderate radical scavenging ability under a lower dosage. However, the observed difference between the model and the GOPs group was not obvious in this study. This result may be explained by the fact that the level of exogenously supplied and endogenously generated ROS is overwhelming and beyond the capacity of natural antioxidant GOPs. Furthermore, in several studies, the peptides isolated from egg, milk, and plant have been identified to have free radical scavenging abilities [37]. Further analysis showed that GOPs strongly enhanced the antioxidant enzyme system and tended to reduce MDA production during the senescent process in NIH/3T3. This also accords with our earlier observations, which showed that GOPs significantly increased antioxidant enzyme SOD and GSH-px activities and decreased MDA content in the liver, pancreas, and muscle of mice under various pathophysiological conditions [15,16,18]. In addition, bioactive peptides extracted from different animal and plant proteins were proven to have antioxidative activity [38]. Overall, bioactive peptides work through a variety of mechanisms to exert their antioxidant properties, but it is mainly completed through delivering hydrogen atoms or electrons to engage in free radical scavenging processes and suppressing free radical production by chelating metal ions [39]. Second, antioxidant enzymes are critical targets for peptide activity in cells and organisms [35]. As reported previously, smaller molecular weight peptides are more likely to approach the free radical reaction center to complete the oxidation reaction chain, and short peptides with fewer than 8 amino acids exhibit substantial antioxidant properties [40]. GOPs are oligopeptides with molecular weights of less than 1000 Da, which serve as the structural foundation for their antioxidant mechanisms. In contrast, peptides with larger proportions of polar amino acids typically exhibit stronger antioxidant activity since the chelation of side chains suppress free radical oxidation [39]. The polar amino acid level of the GOPs was approximately 71.90%, further confirming the strong antioxidant activity of GOPs.

In addition to cell cycle arrest, the induction of a SASP is also one major characteristic of cellular senescence [41]. SASP factors, such as IL-1 and IL-6, trigger paracrine senescence in neighboring areas to reinforce senescence in tissues [42]. We showed that GOPs supplementation significantly inhibited the secretion of IL-6, MMP-3, ICAM-1, and VCAM-1 in NIH/3T3. This could be one of the crucial mechanisms for GOPs to combat cellular senescence. The proinflammatory transcription factor NF-κB is largely responsible for the SASP, which is a transcriptional program to a large extent [43]. We confirmed that the GOPs administration suppressed NF-κB activation. This may, at least in part, account for the mechanism of GOPs exerting their anti-inflammatory effects. Similar observations were made in our previous in vivo experiments, which showed that GOPs inhibited the secretion of cytokines such as IL-1, IL-6, and TNF-𝛼 by downregulating NF-κB in the mouse models of varied degrees of inflammation [15,18]. Furthermore, several reports have shown that bioactive peptides isolated from soybean, sea cucumber, and fish possess prominent anti-inflammatory potential [44,45,46]. In accordance with the assumption that genomic instability is a fundamental driver of the SASP, the key catalyst for NF-kB stimulation is DNA damage response [47,48]. We speculate that the anti-inflammatory potential of GOPs may be partly attributable to its DNA protective effect as mentioned above. Meanwhile, there exists a feedback loop in which SASP expression activates ROS production and DNA damage response [49]. Our findings suggest that GOPs regulate all links of this feedback loop, thus combating senescence progression from multiple dimensions.

Previous studies in our laboratory have shown that GOPs can improve mitochondrial function of skeletal muscles in mice by increasing the mitochondrial DNA content and promoting the mRNA expression of NRF-1 and TFAM [16]. Mitochondria are the essential component in the control of aging. The mitochondrial theory of aging hypothesizes that aging-related sustained mitochondrial dysfunction leads to increased generation of ROS, which in turn aggravates mitochondrial degradation and cellular damage [48]. Thus, we focused on how GOPs affected mitochondrial activity. The results of this study showed that GOPs supplementation improved mitochondrial function as measured by the increased mitochondrial membrane potential in NIH/3T3. In accordance with the present results, numerous investigations have indicated that bioactive peptides derived from marine food can repair or potentiate mitochondrial function after exposure to external stimuli such as H_2_O_2_ and UV radiation [50,51,52]. Aging-associated mitochondrial dysfunction is normally accompanied by impaired turnover in mitochondria owing to decreased biogenesis and clearance [48]. NAD^+^/SIRT1/PGC-1𝛼 signaling pathway, which is involved in mitochondrial biogenesis, is a classical longevity-regulating pathway [53]. We found that GOPs supplementation strongly enhanced NAD^+^ and NAD^+^/NADH activity while up-modulating the protein expression of PGC-1𝛼 in NIH/3T3. Meanwhile, the relative protein expression of SIRT1 was decreased in the model group compared with the GOPs-treated group. Given that SIRT1 needs NAD^+^ to function, this result may be explained by the observation that NAD^+^ depletion decreased SIRT1 consumption in the model group and resulted in a relatively higher level of SIRT1 expression. Lin and Feng found that bioactive peptides isolated from cereals such as corn and potato can promote mitochondrial biogenesis through upmodulating PGC-1𝛼 expression [54,55]. Bioactive peptides can be used as a protein substitute in the daily diet while ensuring amino acid requirements are met. Several studies involving amino acid metabolism during aging also confirmed the correlation between amino acid supplementation and mitochondrial biogenesis. Romano and colleagues reported that the essential amino acid supplementation in mice increases their life span while also promoting mitochondrial biogenesis at the molecular level [6]. Several clinical studies have also shown that branched-chain amino acid supplementation may lessen sarcopenia in the elderly by boosting mitochondrial biosynthesis [2]. In addition, researchers also have found that other biologically active constituents of ginseng, such as ginsenoside, can stabilize mitochondrial membrane potential, increase intracellular ATP production, and enhance mitochondrial biogenesis by activating NRF-1, TFAM, and PGC-1𝛼 [56,57].

Overall, the main finding of this study is that GOPs delayed oxidative stress-induced NIH/3T3 senescence via antioxidant and anti-inflammatory activities and the promotion of mitochondrial biogenesis. These results provide further support for the hypothesis that GOPs may have a positive influence on prolonging lifespan and health span via combating cellular senescence, oxidative stress, and inflammation and protecting mitochondria. Compared to in vitro models, lifespan experiments on mice are more expensive and time-consuming. The current investigation offered a scientific basis for the benefit of long periods of in vivo studies in the future. The results also provide a basis for continued studies to better understand the bioactivity of GOPs. Moreover, evidence now suggests a correlation between oxidative stress and inflammation and chronic diseases like diabetes, hypertension, and atherosclerosis. As a consequence, anti-inflammatory and antioxidant therapies have emerged as potential new approaches to monitor, prevent, and treat chronic diseases [58]. GOPs may have promising applications in this field.

A limitation of this study is that oxidative stress-induced premature senescence cannot fully simulate the natural cellular senescence process. Subsequent primary cell cultures combined with in vivo experiments will offer more conclusive proof. Further research is required to establish the precise exposure dose of GOPs to the cell and to fully understand the effects of GOPs on cell metabolism and the related physiological functions. More efforts will be required to clarify the role of GOPs in prolonging lifespan and health span.

## 5. Conclusions

We have demonstrated that GOPs delayed oxidative stress-induced senescence of NIH/3T3 through the inhibition of cell cycle arrest, promotion of DNA synthesis in the S phase, downregulation of p16^INK4A^ and p21^Waf1/Cip1^ expression, stimulation of cell proliferation, protection of DNA, and promotion of TE activity. Further investigation revealed that the underlying mechanisms of GOPs-delayed senescence were associated with its antioxidant activity, anti-inflammatory effect via downregulation of NF-𝜅B, and promotion of mitochondrial biogenesis via NAD^+^/SIRT1/PGC-1𝛼 pathway. These findings lend support to the hypothesis that GOPs may have a positive influence on extending lifespan and health span via combating cellular senescence, oxidative stress, and inflammation, and protecting mitochondria.

## Figures and Tables

**Table 1:** The Amino Acid Composition of Ginseng Oligopeptides (GOPs).

Amino Acid	Content (g/100 g)	Amino Acid	Content (g/100 g)
Aspartic Acid	0.19	Cystine	0.01
Glutamic Acid	0.12	Valine	0.06
Serine	0.02	Methionine	0.02
histidine	0.06	Phenylalanine	0.09
glycine	0.02	Isoleucine	0.04
Threonine	0.05	Leucine	0.08
Arginine	2.26	Lysine	0.06
Alanine	0.13	Proline	0.65
Tyrosine	0.09		

Table 1 quoted from reference [15].

## Data Availability

The data presented in this study are available on request from the corresponding author. The data are not publicly available due to privacy.
